# Respiratory Constraints in Verbal and Non-verbal Communication

**DOI:** 10.3389/fpsyg.2017.00708

**Published:** 2017-05-17

**Authors:** Marcin Włodarczak, Mattias Heldner

**Affiliations:** Department of Linguistics, Stockholm UniversityStockholm, Sweden

**Keywords:** breathing, multiparty conversation, speech production, multimodal feedback, respiratory planning

## Abstract

In the present paper we address the old question of respiratory planning in speech production. We recast the problem in terms of speakers' communicative goals and propose that speakers try to minimize respiratory effort in line with the H&H theory. We analyze respiratory cycles coinciding with no speech (i.e., silence), short verbal feedback expressions (SFE's) as well as longer vocalizations in terms of parameters of the respiratory cycle and find little evidence for respiratory planning in feedback production. We also investigate timing of speech and SFEs in the exhalation and contrast it with nods. We find that while speech is strongly tied to the exhalation onset, SFEs are distributed much more uniformly throughout the exhalation and are often produced on residual air. Given that nods, which do not have any respiratory constraints, tend to be more frequent toward the end of an exhalation, we propose a mechanism whereby respiratory patterns are determined by the trade-off between speakers' communicative goals and respiratory constraints.

## 1. Introduction

As soon as the focus of a speech researcher's attention shifts from lab setups organized around read speech toward more interactive settings involving spontaneous conversation, one is struck by how many speech phenomena go otherwise unnoticed. While this is both an obvious and seemingly uninteresting observation, the somewhat unexpected upshot of this shift of focus is recasting of familiar problems in novel and often unexpected ways.

In this paper, we would like to perpetrate one such shift with respect to an old-standing problem of respiratory markers of speech planning. While a positive correlation between inhalation depth and duration of upcoming speech has been reported by some studies, both the universality and the strength of this phenomenon has been repeatedly challenged. We propose that the mixed evidence is partly due to not giving enough credit to conversation-specific phenomena, such as verbal and gestural feedback. We hope to demonstrate that by including these elements, the effects are not simply reproduced but rather *reinterpreted* and the puzzle becomes simpler rather than more difficult.

Specifically, we propose that the interaction of breathing and speech does not simply rest on ensuring that the inhalation depth is tailored to an arbitrary linguistic plan. Rather, it is a system of coordinative processes guided by an economy principle, whereby the linguistic plan itself is affected by speaker's communicative intentions and respiratory state. We support this claim by investigating where communicative behaviors with diverging respiratory constraints are initiated within the respiratory cycle. We contrast longer stretches of speech with very short utterances and nods, and demonstrate that as respiratory constraints decrease across the three types, there is a tendency for initiating them later within the respiratory cycle. In addition, we note that gestural feedback expressions are most likely toward the very end of the exhalation and least likely right before exhalation onset. The observed patterns hint at a mechanism whereby verbal backchannels are preferred over purely gestural ones. However, gestural feedback is favored if the speech production costs associated with a new inhalation become too high. We believe the observed patterns are an argument in favor of embodied models of language production.

### 1.1. Respiration and speech planning

Studies on the relationship between respiration and speech planning can be grouped into two related categories. The first is concerned with how breathing is tailored to fit syntactic structure of speech. The second addresses the problem of anticipating longer utterances by taking deeper breaths (cf. Grosjean and Collins, 1979)

Results of studies in the first category have shown that in read speech inhalations coincide largely with syntactic breaks. In fact, Lieberman (1967) defined the term *breath group* purely in terms of prosodic-syntactic (rather than physiological) criteria as a typical intonational pattern over declarative sentences. In the same study, he found that nearly 90% of all sentences were produced on a single breath. Other studies of read speech reported even higher percentages of inhalations coinciding with syntactic boundaries: 96.8% (Winkworth et al., 1994), 98.2% (Wang et al., 2010), and 100% (Henderson et al., 1965).

In addition, the likelihood of an inhalation was observed to depend on the strength of syntactic break and on speech tempo. Grosjean and Collins (1979) found that at slower speaking rates (around 75 words per minute) inhalations coincided mainly with major syntactic boundaries but also to some extent with minor breaks. As the rate increased, however, inhalations at minor breaks disappeared, and at very high rates pausing was predominantly controlled by physiological demands rather than by syntactic chunking. Similarly, Conrad et al. (1983) reported higher frequencies of inhalations at stronger syntactic and/or textual breaks (paragraphs, sentences) than at weaker ones. In addition, syntactically weaker positions were associated with shallower inhalations. Similarly, Bailly and Gouvernayre (2012) demonstrated that breathing is used for marking thematic structure of read texts.

By contrast, studies of spontaneous and/or conversational speech have shown much higher numbers of inhalations occurring inside syntactic constituents: 13% (Wang et al., 2010), 31% (Henderson et al., 1965), 15.3% (Winkworth et al., 1995). The difference was attributed largely to increased cognitive effort of extemporaneous speech production (Mitchell et al., 1996).

Studies in the second category are inspired by “[a] natural expectation […] that longer utterances should be preceded by longer inspirations” (Whalen and Kinsella-Shaw, 1997, p. 138). The respiratory anticipation is hypothesized to ensure that speakers have enough air to produce the upcoming utterance without going below the *resting expiratory level* (REL), that is the lung volume at which the elastic recoil forces of the thorax and the lungs counter each other.

This issue has been revisited by numerous studies. Overall, they have found a relationship between utterance duration and/or inhalation depth both in read speech (Winkworth et al., 1994; Whalen and Kinsella-Shaw, 1997) and in spontaneous conversational speech (Winkworth et al., 1995; Rochet-Capellan and Fuchs, 2013). However, the evidence in favor of respiratory anticipation of the upcoming utterance is by no means universal. For instance, Autesserre et al. (1989) and Guaïtella (1993) failed to find a correlation between inhalation depth and utterance durations, and Horii and Cooke (1978, p. 477) concluded that “typically, oral reading is done well within a respiratory capability (equilibrium) and does not usually require special modification of respiratory maneuvers that are dependent on the length of subsequent utterance. These data thus support a notion of semi-independence of the respiratory system to speech production such that depth of inspiration is unrelated to the subsequent utterance length, at least in the oral reading task.” On that view, precise planning is not necessary as speakers can always compensate for it by speaking on the expiratory reserve volume (i.e., the volume below REL). Similar conclusions were reached by Hoole and Ziegler (1997), who found only a limited effect of utterance duration on the magnitude of the preceding inhalation compared to the difference in exhaled air volume. In effect, while shorter utterances were produced at lung volumes above REL, longer utterances usually infringed on the expiratory reserve volume. In addition, several studies have attempted to find a relationship between inhalation parameters and the syntactic complexity of the upcoming utterance, but the results were mixed (Whalen and Kinsella-Shaw, 1997; Rochet-Capellan and Fuchs, 2013; Fuchs et al., 2015a).

Finally, while statistically significant, the effect of utterance duration accounted for a relatively small part of the total variance and often showed large between-subject variability. For instance, as shown by Denny (2000), much of the cycle-to-cycle variability is unrelated to speech preparation but can instead be attributed to the same control mechanisms, which result in comparable variability during quiet breathing.

In conclusion, the problem of coordination of speech and breathing can hardly be claimed to have been solved once and for all. Notably, the reported effects were relatively weak and speaker-dependent. More importantly, however, the material used in the studies was not representative of spontaneous conversational speech. If spontaneous speech was elicited at all, it included a monolog (Rochet-Capellan and Fuchs, 2013) or a conversational task in which the interlocutor was the experimenter who attempted “to maximize the number of the subject's utterances by providing appropriate questions and prompts” (Winkworth et al., 1995, p. 127).

### 1.2. Speech respiration and economy principle

Most of the work summarized in the previous sections views the interaction between speech and breathing as being driven by linguistic planning, which in turn determines breathing patterns observed in speech production. The view rests on deeply ingrained concepts about speech production, whereby linguistic planning is an autonomous process carried out in a disembodied fashion. On that view, motor planning is largely determined and subordinate to linguistic planning and is limited to execution of the latter with no feedback loop between the two systems.

In this paper we propose to abandon the view of the relationship of speech and respiration as a one-way execution pathway. Instead, we are interested in whether and how the respiratory state itself shapes speech production. We submit that linguistic planning does not always unconditionally override the ongoing breathing activity. Rather, speaker's communicative intentions are compared against respiratory effort associated with producing an utterance, which we link to initiating a new respiratory cycle. Coordinating speech and breathing can be thus seen as an optimization problem and should follow the economy principle, which is a pervasive mechanism in speech production (Lindblom, 1990).

Specifically, in his sketch of the H&H theory, Lindblom (1990) proposed a model of output-oriented (or goal-driven) speech production in which output constraints are weighted against constraints of the production system. In the process, the target production (the *should-be*) is compared against the momentary state of the system (the *is*), and the optimal realization is selected depending on the admissible discrepancy between the two. By default, system constraints dominate and the system gravitates toward the low-cost solution. At the same time, system constraints can be overridden by compensatory activity to reach the prescribed articulatory target. The trade-off between the output and system constraints is thus listener-oriented: extra production costs can be incurred to ensure sufficient discriminability and, in consequence, successful communication.

While the H&H model was in the first place proposed to account for the problem of variability in the speech signal, it can be easily translated into the domain of speech breathing. In the present paper, we compare how longer stretches of speech and feedback expressions (both verbal and non-verbal) are timed with respect to the respiratory cycle. If the H&H model correctly describes the coordination of speech and breathing, we should see different temporal patterns across the three types of communicative behavior reflecting their respiratory requirements.

In particular, short verbal feedback expressions (SFEs, such as “mhm,” “aha,” “ja”) are shorter and quieter than proper dialogue turns, which in turn contributes to their unobtrusive nature (Heldner et al., 2010). Due to these properties, SFEs are likely to have modest respiratory requirements and can be in principle produced even on low lung volumes, thus not requiring a new inhalation. Consequently, we expect SFEs to be distributed more uniformly within the respiratory cycle. By contrast, longer stretches of speech are expected to follow an inhalation directly. This is in line with the economy principle: given that production effort is mainly associated with starting a new inhalatory cycle (Aleksandrova and Breslav, 2009), using residual air minimizes production costs while realizing speaker's communicative intentions.

In addition to verbal feedback, we investigate one important type of non-verbal feedback, head nods, which fulfill similar communicative functions (see Wagner et al., 2014 and references therein), but are completely free from physiological respiratory constraints. We predict non-verbal feedback to be produced more frequently toward the very end of the respiratory cycle, when the respiratory requirements of even a short verbal feedback expression cannot be met. In other words, we predict that when speakers run out of air, they choose a lower-cost functional equivalent of a verbal expression.

It is worth noting that while feedback expressions are both ubiquitous and essential for interspeaker coordination in spontaneous conversation (Duncan and Fiske, 1977), very little is known about their respiratory characteristics. In fact, in most earlier studies (e.g., McFarland, 2001; Rahman et al., 2011; Rochet-Capellan and Fuchs, 2014) backchannels were included in the “quiet breathing” category, a decision reflecting the canonical definition of backchannels as dialogue contributions which do not claim the conversational floor (Yngve, 1970) but not motivated by their respiratory properties. A notable exception is Rochet-Capellan et al. (2014), but backchannels were outside the main focus of their analysis.

The hypothesized effect of respiratory requirements on temporal coordination of SFEs was borne out by a preliminary study of dyadic Estonian interactions (Aare et al., 2014). The study found that backchannel-like utterances were indeed more likely to be initiated later in the respiratory cycle than non-backchannel turns, indicating that they might require less respiratory planning than evidenced in longer stretches of speech. Notably, Rahman et al. (2011) reported that backchannels occurring during periods of listening results in larger cycle amplitudes than those found in silent breathing. However, it is possible that the effect was due to increased exhalation amplitude while vocalizing rather than due to deeper inhalations. In addition, a recent study of breathing patterns in question-answer sequences (Torreira et al., 2015) reported that an inhalation is more likely to occur directly before long answers than before short ones.

## 2. Materials and methods

The results in this study were drawn from eight three-party conversations recorded in a sound-treated studio in the Phonetics Laboratory at Stockholm University. The conversations were on average 23 min in duration and the total duration of the eight recordings was 3 h 5 min.

The participants were 12 males and 12 females (median age = 25 years; IQR = 23–27 years). They were all native speakers of Swedish and most of them were students or staff at universities in the Stockholm area. Half of the conversations included two females and one male, the other half two males and one female. With the exception of two conversations, all speakers knew each other prior to the recording. The participants volunteered to participate in the study, gave their written informed consent before the recording, and were reimbursed for their participation with one cinema ticket each.

**Figure 1:**
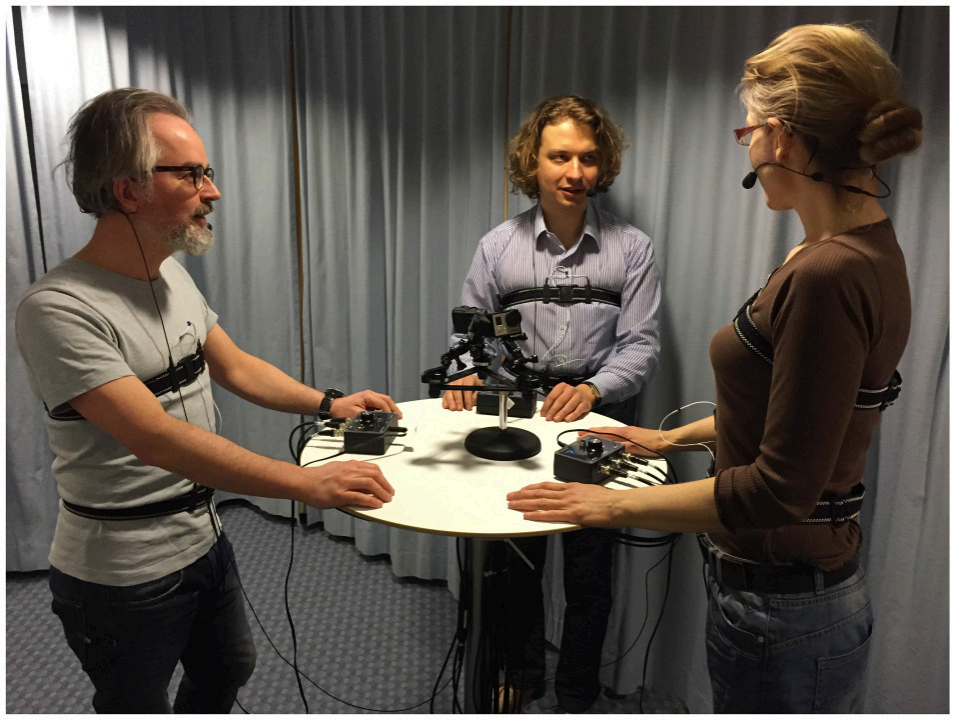
**Recording setup with mock subjects around a bar table wearing Respiratory Inductance Plethysmography (RIP) belts connected to RespTrack processors and microphones**. Video cameras are placed on the table. Reproduced from Włodarczak and Heldner (2016a) with the permission of ISCA and published with subjects' written informed consent.

The participants were recorded standing around a bar table (height 105 cm) to avoid changes in the breathing pattern due to sitting posture shifts (Lee et al., 2010). The topic and the course of the conversations was not restricted in any way. The participants were instructed to talk about absolutely anything they wanted at any point during the recording. However, they were asked to avoid large arm and torso movements, which would otherwise distort the respiratory traces. The recording setup is shown in Figure 1.

Each participant's respiratory activity was recorded using *Respiratory Inductance Plethysmography* (RIP, Watson, 1980). This technique measures changes in cross-sectional area by means of elastic belts with zigzagging (coiled) wires sewn into them. One belt is worn around the chest (at the armpit level) and another around the abdomen (at the navel). The belts are connected to a processor that generates a weak magnetic field and measures the opposing current created by changes in cross-sectional area resulting from inhalations and exhalations. Much previous research in this field has used the Respitrace system from Ambulatory Monitoring Inc. In our setup, we used commercially available RIP belts (Ambu RIPmate, pediatric size) connected to a RespTrack processor, developed in-house in the Phonetics Laboratory at Stockholm University. We designed our own processor as the one supplied with the belts included hardwired high-pass filters which made it impossible to distinguish between breath-holding and slow exhalations. The RespTrack processor transforms the respiratory movements of the rib cage and abdomen into direct voltages in the range −2*V* to +2*V*. In addition, it features an output with the weighted sum of the two inputs corresponding to total lung volume. The belts are connected to the processor via isolation transformers and high impedance resistors in the connection cable.

**Figure 2:**
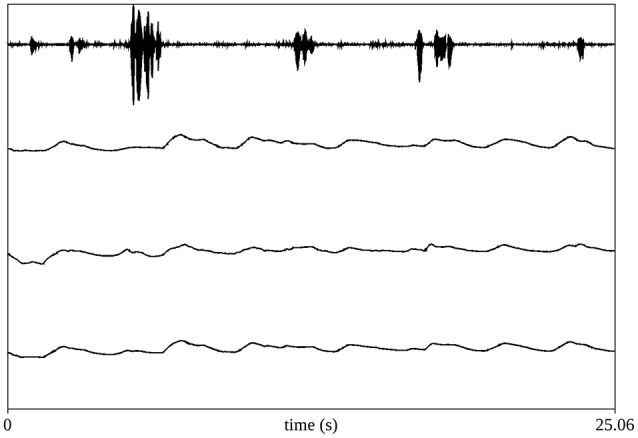
**Sample respiratory and speech signals from one speaker**. The top channel (channel 1) contains speech; channels 2 and 3 contain respiratory measurements from the rib-cage and abdomen RIP belts; the bottom channel shows the weighted sum of the two belts.

The respiratory signals were captured by an integrated physiological data acquisition system (PowerLab hardware and LabChart software by ADInstruments) at 1 kHz sampling rate, 16 bits per sample. Figure 2 shows sample respiratory and speech signals for one participant. Before the conversations the sensitivity of each belt was estimated by means of the isovolume maneuver (Konno and Mead, 1967). Vital capacity and resting expiratory level (REL) were also estimated (Hixon et al., 2014).

Speech was collected using close-talking microphones with a cardioid polar pattern (Sennheiser HSP 4), an audio interface (Motu 8M), and a digital audio workstation (Reaper). Audio was stored in uncompressed format, 48 kHz sample rate, 16 bits per sample. In addition, lower quality audio was routed to PowerLab to allow synchronization with the respiratory signals. Three GoPro Hero3+ cameras placed on the table captured speakers' heads and torsos. All recording devices (including the three video cameras) were post synchronized in FinalCut Pro X using the audio recorded by the different devices.

The research was approved by the Regional Ethical Committee in Stockholm (2015/63-31), and all appropriate permissions have been obtained from the copyright holders of any work that has been reproduced in this work. We have also obtained written consent from participants portrayed in Figure 1 for publishing the photograph.

### 2.1. Measures

**Figure 3:**
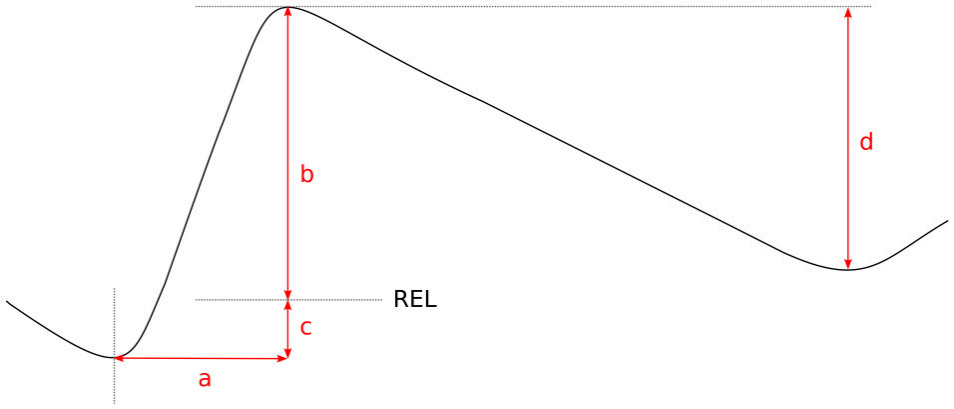
**Parameters of the respiratory cycle: inhalation duration (a), inhalation amplitude with respect to REL (b), amplitude ratio ([*b*+*c*]/*d*)**.

In order to identify respiratory cycles, the summed signal from both belts was normalized by replacing each sample by a rolling *z*-score within a 10-s window and the minima and maxima which were at least 1 standard deviation apart were then taken as inhalation onsets and offsets. The following parameters relevant to the planning hypothesis were subsequently extracted for each cycle: (1) inhalation duration, (2) inhalation amplitude with respect to REL, and (3) inhalation-to-exhalation amplitude ratio (see Figure 3). The reason for measuring inhalation amplitude above REL is to remove the effect of the previous cycle, which, especially when it coincides with a longer utterance, may be completed below REL. We also evaluated the degree of planning by including the inhalation-to-exhalation amplitude ratio, which under perfect planning was expected to equal 1 (0 on log-scale). The ratio was calculated using the whole inhalation amplitude. While using the REL-corrected values might have been preferable, it resulted in high colinearity with the other measures and complicated statistical modeling.

Inhalation duration was expressed in log_2_ ms. Inhalation amplitude above REL was expressed as percentages of speaking volume, whose limits were estimated at the 2th and 98th percentiles of speaker's respiratory values. Given that REL is heavily influence by posture shifts, it was estimated following a procedure we proposed in Włodarczak and Heldner (2016b). Specifically, REL was taken as the mean level of troughs in the previous 20 respiratory cycles. The amplitude ratio was also log-transformed to remove the skew.

Speech and silence segments in the speech signal were annotated semi-automatically by manual correction of intensity-based voice activity detection in ELAN (Wittenburg et al., 2006). Speech segments shorter than 1 second were classified as *very short utterances* (VSUs) and longer ones as *SPEECH*. The VSU class was proposed by Edlund et al. (2010) to overcome problems with defining and identifying backchannel-like short feedback expressions in conversation. They evaluated the method on Columbia Games Corpus, a large corpus of task oriented dialogues with manual annotations of utterance functions. They found that the category of utterances shorter than 1 second captured all backchannels, which comprised 31% of all VSU. Another 40% of VSUs were *affirmative cue words* (short lexical items communicating agreement, such as *alright, yes, yeah*, etc.) and the 25 most frequent tokens of the remaining 29% of VSUs were also used to indicate feedback (e.g., *cool, got it, mm*, etc.). Hence, VSUs correspond predominantly to short feedback expressions that were of interest here. Participant produced on average 9.3 VSUs per minute (sd = 3.3).

Nods (head movement along the midsagittal plane) were manually annotated in ELAN. The direction of movement (upwards or downwards) and the number of cycle repetitions were not labeled. Nodding data was obtained for 19 of the speakers. The video was unavailable for three speakers due to a technical error. Two further speakers were excluded due to difficulties in segmenting their head movements. Mean nod rate across participants equaled 3.8 nods per minute. There was substantial variation across the speakers in the number of nods produced per minute (sd = 2.9). Speakers also differed in the number of VSU to nods produced: the average VSU to nod ratio equaled 3.6 with the standard deviation of 2.4.

Similar to other breathing studies (Fuchs et al., 2015a), we identified and excluded segments of laughter from the data. Laughter was detected automatically using a version of the algorithm described by Urbain et al. (2013) based on (z-scored) velocity and acceleration profiles. Manual inspection of the output of the laughter detector indicated that the method resulted in some false positives. However, as we were only using this technique for *data filtering*, this simply resulted in a smaller analyzed sample.

We also excluded instances of inhalations coinciding with speech. Although it is certainly possible to produce speech, and especially short feedback expressions on an ingressive air stream in Swedish, manual inspection of the video recordings indicated that most of the inhalations coinciding with speech here were artifacts due to gesturing or posture shifts, or were errors in the annotation.

By combining these measures, we extracted two different data sets. First, we combined information about speech and silence segments with the respiratory cycles to classify respiratory cycles according to what happened in them. Thus, we identified respiratory cycles in silent breathing (SILENT), in speech breathing (SPEECH), and in VSU breathing (VSU). The results in Section 3.1 were based on this data. The data set in this section included 2,921 SILENT cycles, 969 SPEECH cycles and 1,426 VSU cycles.

**Figure 4:**
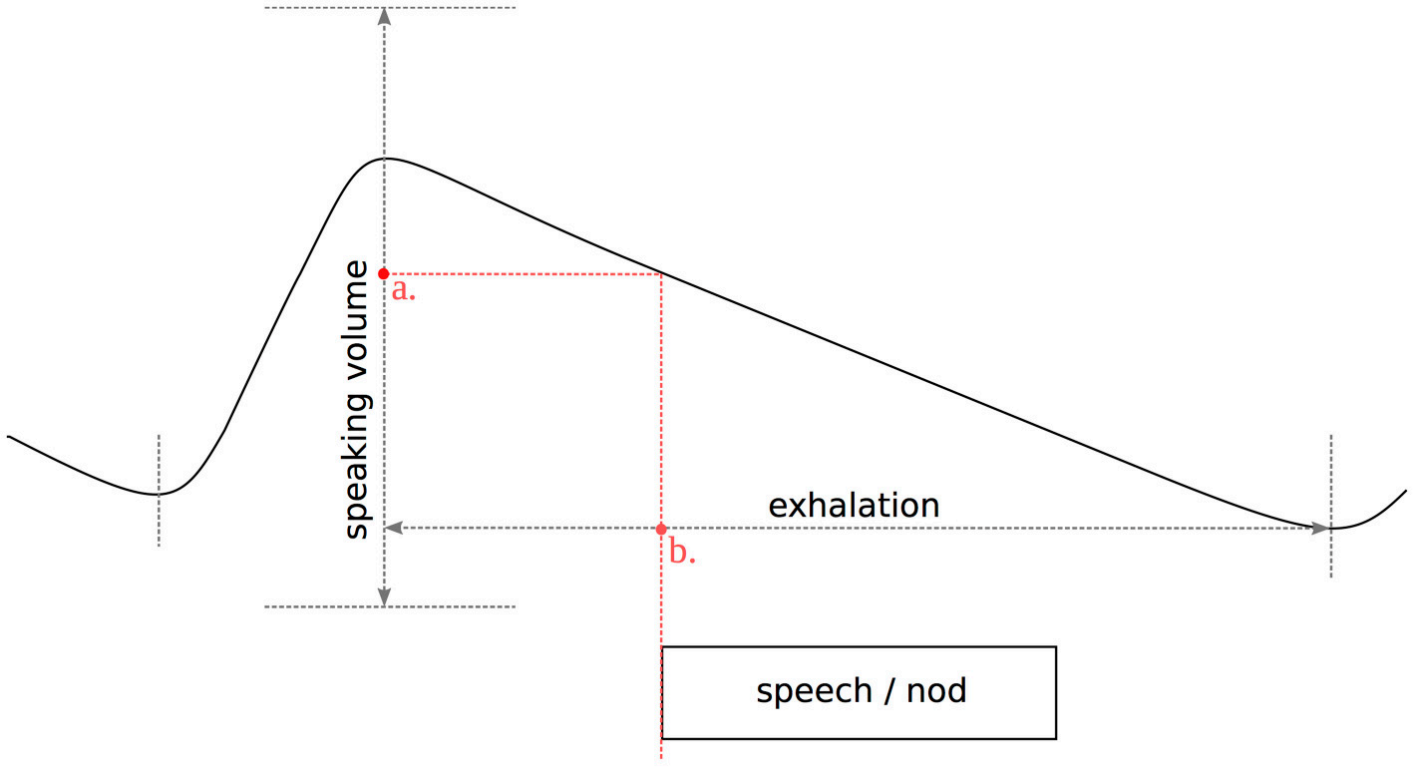
**Position of a speech or nod segment onset with respect to (a) speaking volume, and (b) exhalation duration**. Reproduced from Włodarczak et al. (2015) with the permission of ISCA.

Subsequently, we described different types of communicative behavior in terms of where they occurred in the respiratory cycle. The onsets of VSUs, SPEECH segments and nods (both occurring on their own and overlapping with a VSU) were normalized relative to: (1) their position within exhalation duration, and (2) their position within speaking volume (see Figure 4). If two speech segments or nods occurred in one cycle, this resulted in two data points. The resulting values were expressed as the fraction (with values between 0 and 1) of exhalation duration and speaking volume at which a segment starts, respectively. The results in Section 3.2 were based on this data and included 904 SPEECH cycles, 1,473 VSU cycles, 356 nods, 292 nods coinciding with a VSU (nod+VSU).

## 3. Results

### 3.1. Respiratory cycle parameters

**Figure 5:**
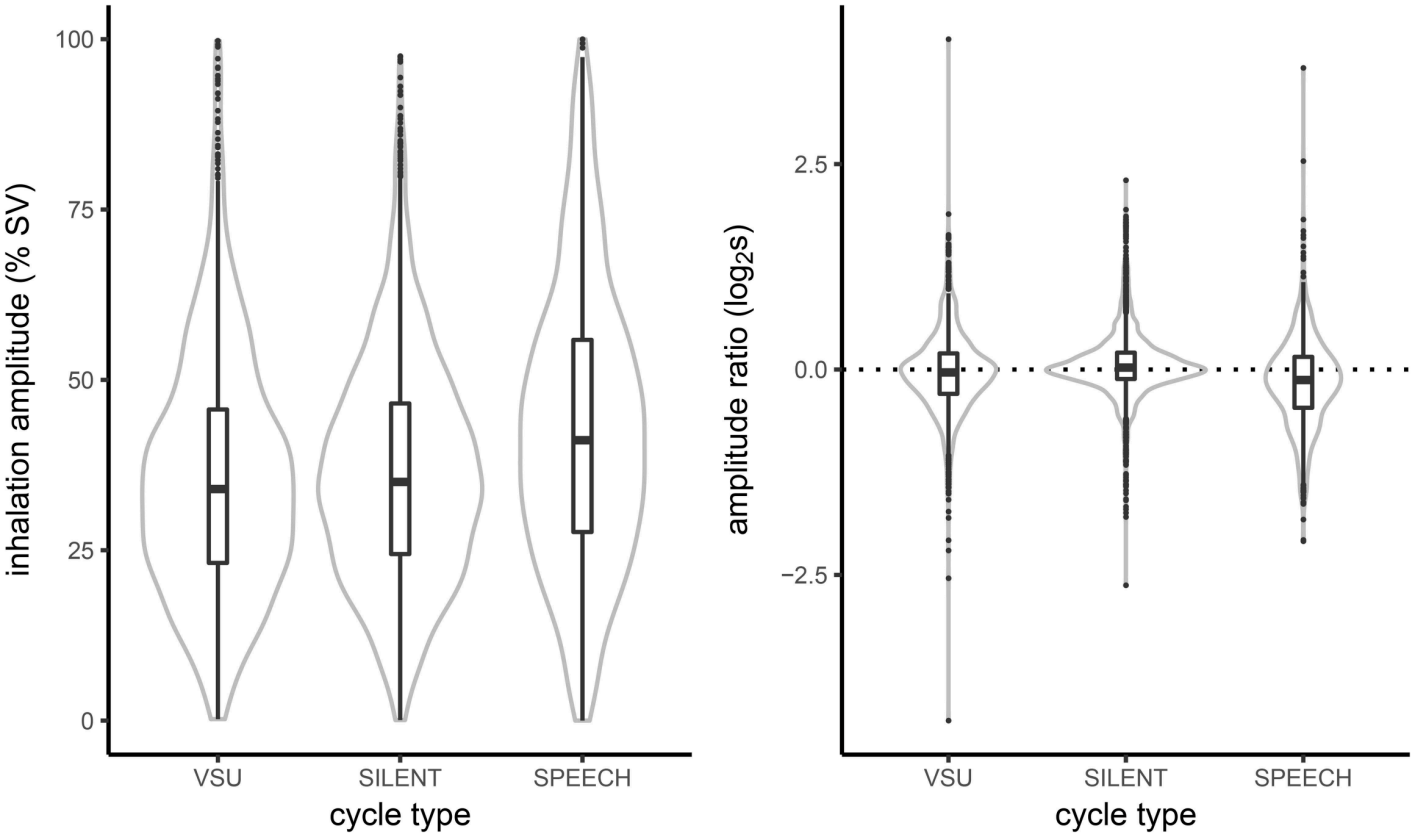
**Kernel density estimates and boxplots of inhalation amplitude (Left)** and inhalation-to-exhalation amplitude ratio **(Right)** in respiratory cycles coinciding with silence, speech and VSUs.

First, we examined how respiratory amplitude varied across cycles in silent breathing, cycles coinciding with VSUs and cycles including speech. In the left panel of Figure 5 we plot kernel density estimates (mirrored around the ordinate) with overlayed box and whiskers plots of inhalation amplitude in the three cycle types. Both the figure and the mean amplitudes across the three cycle types (35.7, 36.4, and 42.9 for VSU, silent and speech cycles, respectively) indicate that VSU cycles were indeed more similar to silent cycles than to speech breathing, which was characterized by substantially greater amplitude. In addition, in our data the three distributions overlapped to a large degree. However, it is likely that the recording conditions did not require lung volumes much larger than the tidal volume. Indeed, the analyzed material consisted of friendly, non-competitive conversations recorded in a quiet laboratory environment with participants standing in close proximity to one another.

Analysis of the inhalation-to-exhalation amplitude ratio, shown in the right panel of Figure 5 also suggests limited planning in the VSU (as well as speech) cycles. Namely, both VSU and speech cycles had negative amplitude ratio means (−0.04, −0.16, respectively), indicating that on average more air is exhaled than had been inhaled (the reader is reminded that amplitude ratio is expressed on log-scale and, consequently, 0 corresponds to 1 on linear scale). By contrast, silent cycles have a slightly positive ratio (0.06). The result suggests that the extra air necessary to produce a backchannel (or speech) was not anticipated at the onset of a breathing cycle.

**Figure 6:**
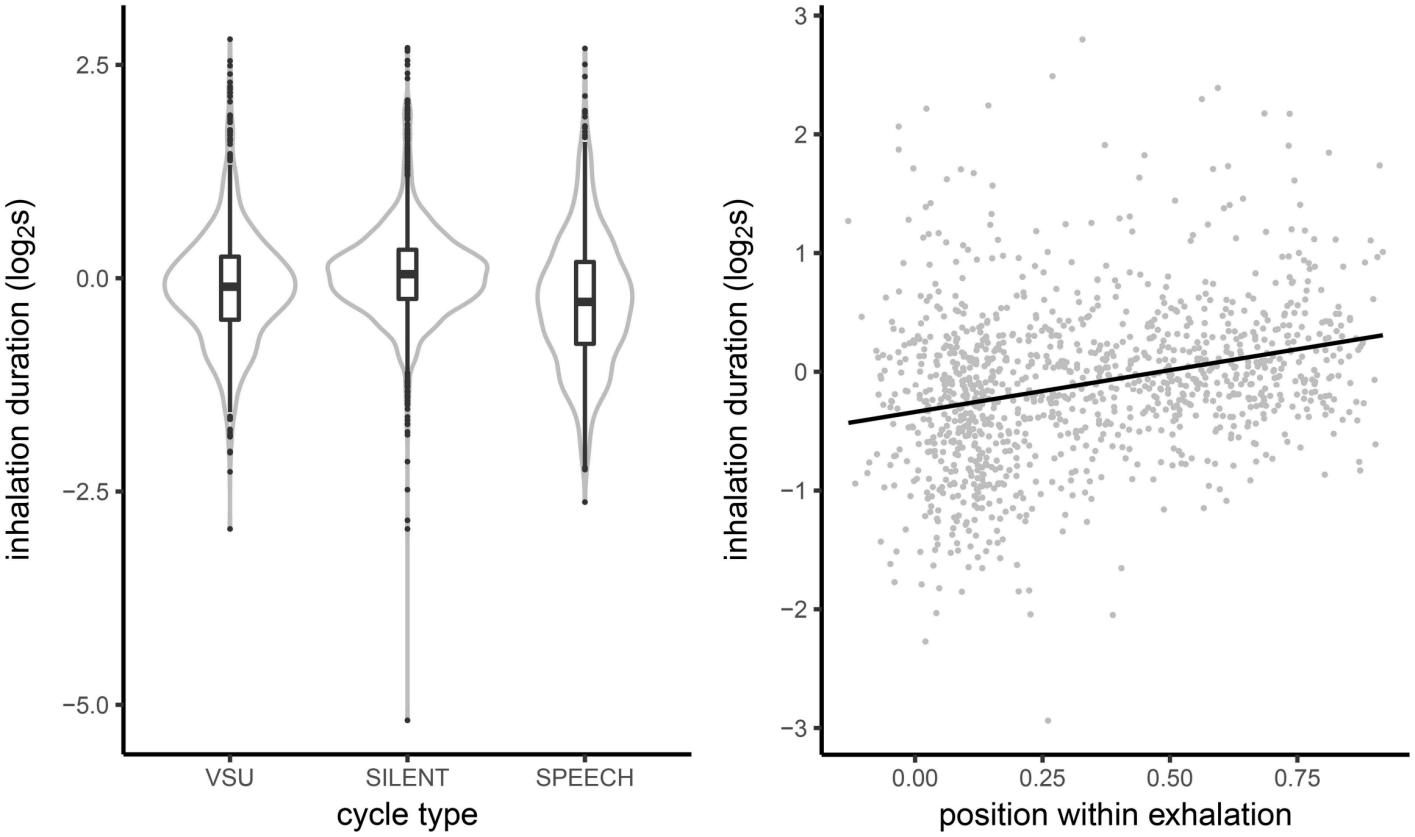
**(Left)** Kernel density estimates and boxplots of inhalation duration in respiratory cycles coinciding with silence, speech and VSUs. **(Right)** The relationship between inhalation duration and relative timing of VSU onsets in the exhalation.

Next, we examined how inhalation durations varied across cycles with silent breathing, cycles including VSUs and speech cycles. Inhalation durations in the three cycle types, plotted in the left panel of Figure 6, show the expected pattern of pre-speech inhalations being shorter than inhalations in quiet breathing (with the means of −0.27 and 0.07, respectively). While much longer than inhalations found in speech, inhalations in VSU cycles were substantially shorter than those in silent breathing (−0.11). To account for the difference, in the right panel of Figure 6 we plot the relationship between inhalation duration and the relative timing of VSU onset in the exhalation. A clear linear relationship can be discerned, suggesting that while VSUs early on in the exhalation may have been to some extent planned, the effect is weaker for short vocalizations late in the exhalation.

The contribution of the three features to predicting cycle type were evaluated by fitting a multinomial logistic regression model. The model was build hierarchically following the procedure outlined in Field et al. (2012). Specifically, the predictors were added one at a time and the improvement of model fit was assessed in terms of reduction of −2 × log-likelihood. Since all three features improved the model significantly (*p* < 0.001), they were included in the final model, summarized in Table 1. As can be appreciated from the table, all features significantly contributed to the cycle type distinction. The effect was particularly pronounced for inhalation duration and amplitude ratio. An increase of inhalation duration by one unit increases the odds for silent cycles by about 0.4 and reduces the odds for speech by 0.5. A one-unit increase in amplitude ratio produced similar change in odds ratios. The effect of inhalation amplitude was only significant for the VSU/speech distinction, whereby an increase of inhalation amplitude by 1 per cent increased the odds for speech by 0.03. The fact that the effect was not significant for the VSU/silent distinction is somewhat at odds with the significant influence of inhalation duration, however the latter can be accounted for by the influence of VSUs occurring early in the exhalation. We address temporal organization of communicative behaviors in more detail in the following section.

**Table 1:** **Coefficients of the multinomial logistic regression model (95% bootstrap confidence intervals for odds ratio based on 1000 iterations)**.

		**B**	**exp(B)**	**95% CI**		***p***
				**LL**	**UL**	
Silent	Constant	0.772	2.164	1.864	2.515	0.00
	Inhalation amplitude	−0.001	0.999	0.995	1.002	0.46
	Inhalation duration	0.440	1.552	1.371	1.733	0.00
	Amplitude ratio	0.416	1.516	1.302	1.763	0.00
Speech	Constant	−1.521	0.218	0.171	0.275	0.00
	Inhalation amplitude	0.026	1.026	1.021	1.032	0.00
	Inhalation duration	−0.472	0.624	0.528	0.732	0.00
	Amplitude ratio	−0.490	0.613	0.498	0.767	0.00

*The reference category is VSU. Model χ^2^(6) = 513.34, p < 0.001, pseudo-R^2^ (McFadden) = 0.05*.

### 3.2. Temporal analysis

**Figure 7:**
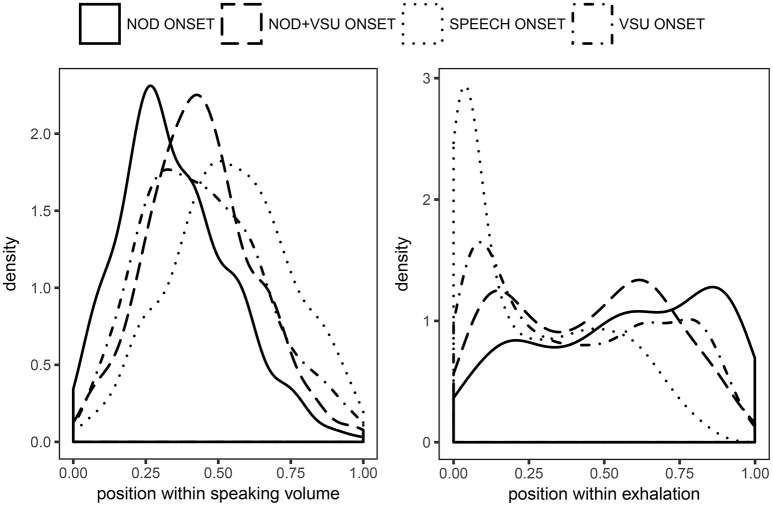
**Kernel density estimates of nod, nod+VSU, speech and VSU segments onset timing relative to speaker's speaking volume (Left)** and exhalation duration **(Right)**.

Following the analyses of inhalation amplitude, inhalation duration and amplitude ratio, we also examined where the onset of speech segments, VSU segments, and nods fell within the respiratory cycle. Kernel density estimates of speech and nod segment onsets normalized to speaker's speaking volume are plotted in the left panel of Figure 7. Accordingly, in that figure the abscissa corresponds to the total lung volume range used by each speaker. As can be seen, nods were initiated at lower lung volumes (on average at 0.35), followed by VSUs and /nod + VSU/ composites (0.44, 0.45, respectively) and speech segments (0.54). The results were thus in line with the reduced respiratory requirements of non-verbal feedback, whether or not accompanied by speech, than for speech-only segments.

The distributions were compared by means of mixed-effects models with speakers entered as random effects and were found to be significantly different [*F*_(3, 3020.6)_ = 64.42, *p* < 0.001]. Pairwise comparisons between segment types using Tukey's HSD test revealed statistically significant differences between all classes (*p* < 0.001), except for *VSU* and *nod*+*VSU* (*p* = 0.52).

A compatible picture can be seen in the right panel of Figure 7, where position of segment onsets is normalized to expiration duration. Predictably, longer speech segments are started predominantly right at the beginning of the exhalation. After that their likelihood dropped sharply, and they were extremely rare in the second half of the expiration. While VSUs also showed a tendency to start toward the beginning of the exhalation, this effect was weaker and they were initiated relatively frequently up to about 75% of the exhalation duration. In addition, and perhaps most interestingly, nods were found more often late in the exhalation than toward its beginning, deviating significantly from the uniform random distribution (one-sample Kolmogorov-Smirnov test, *p* < 0.01). The nod+VSU distribution was clearly bimodal with one peak in the vicinity of exhalation onset and another around 70% of its duration. The distribution of crossmodal nod+VSU segments thus bore much similarity to the summed distributions of unimodal nods and VSUs. Statistical significance between the four distributions was assessed by means of pairwise two-sample Kolmogorov-Smirnov tests with Bonferroni correction to compensate for multiple comparisons. All comparisons were statistically significant at *p* < 0.001, except for *nod*+*VSU* and *VSU* which was significant at *p* < 0.05.

**Figure 8:**
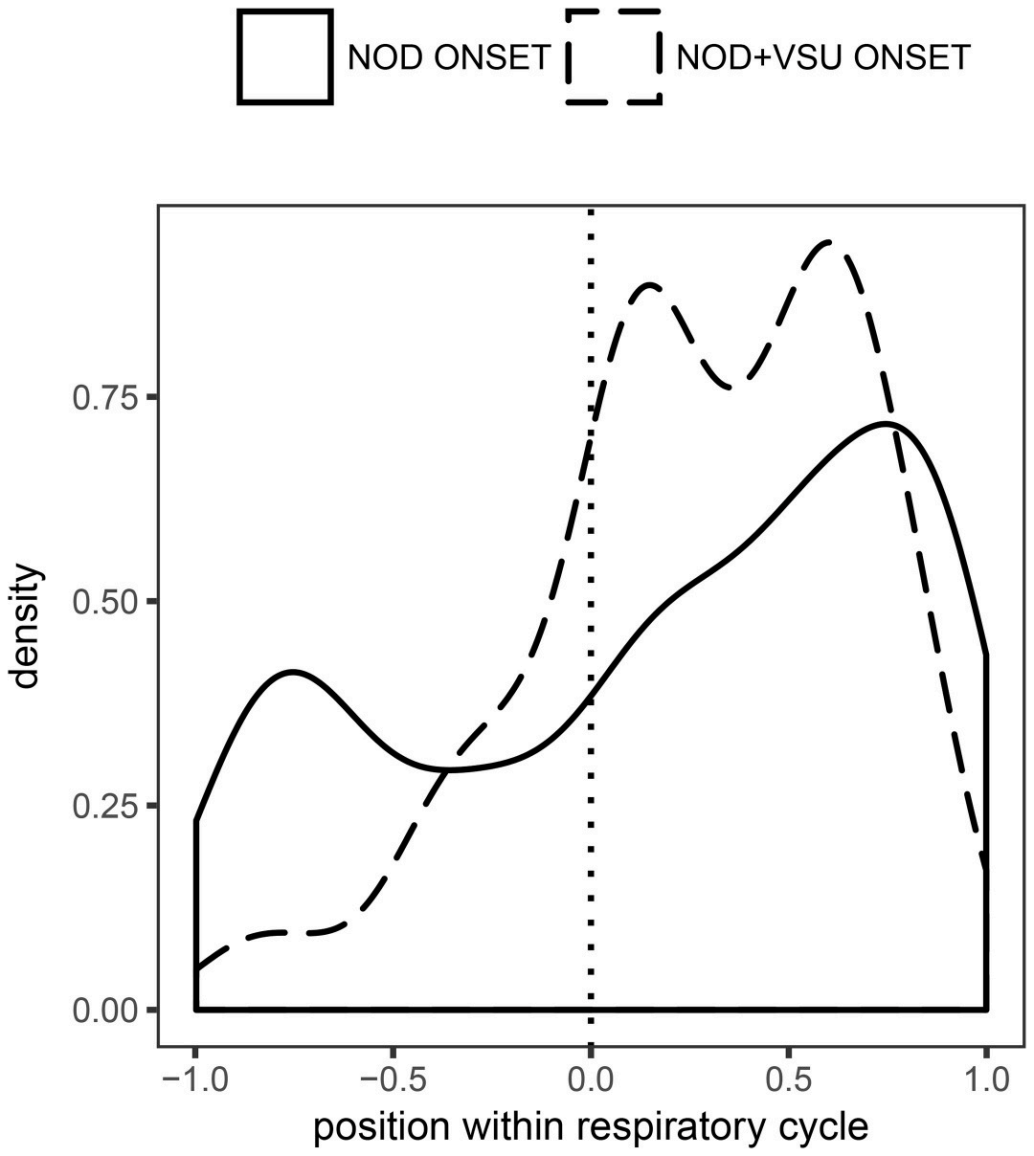
**Distribution of nod and nod+VSU onset timing normalized to breathing cycle duration**. 0 on the abscissa corresponds to exhalation onset, −1 and 1 correspond to inhalation onset and exhalation offset, respectively.

Notably, as nods have no respiratory constraints, they can in principle coincide feely with inhalations. This was indeed the case for 191 instances of nods and 87 instances of the nod+VSU class. Consequently, we then studied temporal organization of nods and nod+VSU composites in the respiratory cycle as a whole by normalizing their relative position in the inhalation to the interval [−1, 0]. In the resulting distribution, presented here in Figure 8, −1, 0, and 1 thus correspond to inhalation onset, inhalation offset and exhalation offset, respectively. While the nod+VSU distribution shows the familiar bimodal pattern (cf. Figure 7), the distribution of nods reaches its maximum near cycle boundaries and minimum directly before the inhalation offset.

## 4. Discussion and conclusion

The aim of this paper has been to challenge the dominant view of coordination between speech and breathing as a one-way execution pathway with linguistic planning determining respiratory patterns in an arbitrary way. Instead, we hypothesized that linguistic planning itself relies on and is shaped by respiratory requirements and speaker's momentary respiratory state. In order to test this hypothesis we compared respiratory patterns in longer stretches of speech, VSUs as well as gestural feedback. We predicted divergent coordinative patterns, which were borne out by the analysis presented above. Specifically, unlike longer utterances requiring more air in the lungs, VSUs, whose respiratory demands are more limited, are distributed much more uniformly within the respiratory cycle and are found up to 75% of the exhalation duration. In addition, dialogue participants were found nodding most frequently toward the end of the exhalation. At the same time, nods were dispreferred when the inhalation was almost complete.

The coordination of speech and breathing thus appears to conform to the economy principle (Lindblom, 1990). Specifically, if the speaker has enough air in the lungs to satisfy the requirements of the upcoming utterance, it is produced on residual breath (cf. Torreira et al., 2015). However, when lung levels become too low for sustaining even a short vocalization, gestural feedback is preferred in place of verbal feedback, as evidenced by the fact that nods are more likely in the vicinity of inhalation onsets. The distributions of segment onsets within the speaking volume provided consistent, if somewhat less clear, evidence. The results are also in accordance with those of McFarland and Smith (1992), who found a range of pre-speech adaptations of the rib cage and the abdomen depending on the momentary lung volume.

An interesting upshot of our results is that verbal feedback and nods are not functionally equivalent. If this were the case, a system driven by an economy principle would gravitate toward the lower-cost nods. That this is not the case suggests that verbal feedback is often preferred at the expense of the additional production cost. However, toward the end of the exhalation, the cost associated with starting the new inhalation most likely outweighs the added pragmatic benefit of the verbal feedback and a nod is produced instead.

Another interesting corollary of our findings is that they help avoid delays in the timing of feedback (cf. Torreira et al., 2015). In particular, using residual air to produce SFEs prevents a lag introduced by an inhalation. Nodding at very low lung levels or while inhaling has a very similar effect. It is a curious observation that nods were the least likely just before the inhalation offset, which indicates that gestural feedback is disfavored when the incurred delay is small enough to be outweighed by the preference for verbal feedback.

The results summarized in Section 3.1 provide further evidence of substantial similarity between respiratory cycles coinciding with VSUs and found during listening periods (i.e., silent cycles). Both listening and VSU cycles are characterized by longer inhalations than those before longer stretches of speech. Consequently, given the small differences in inhalation amplitudes between the two types, we find limited evidence for respiratory planning in VSUs, especially those produced especially for those VSUs produced later in the respiratory cycle. Although inhalation duration preceding VSUs was on average longer than in silent breathing, the effect seems to be largely due to vocalizations produced early on in the exhalation. In other words, whatever planning processes are present, they get weaker for VSUs produced toward the end of the respiratory cycle. This is also in line with the hypothesized low respiratory demands of VSUs, which allow them to be produced at lung volumes approaching REL without much respiratory adjustment. The findings are thus in line with the accounts which stress temporal autonomy of backchannels (Heldner et al., 2010, 2013).

More generally, we have observed little difference in amplitude across the respiratory cycle types. While this is initially surprising, we submit this is a likely outcome of the recording environment, which in our case was a sound-treated recording studio. Unlike in field recordings (Rahman et al., 2011), producing conversational speech in this setting supposedly did not demand inhaling much above the tidal volumes.

Lastly, our findings suggest that crossmodal feedback (i.e., the nod+VSU segments) is not much different from unimodal (purely gestural or purely verbal) feedback. This is apparent from the fact that the temporal distribution of the nod+VSU segments resembled that of the summed distributions of unimodal nods and VSUs. In other words, cross-modal feedback is not special with respect to its position within the respiratory cycle, which might in turn be an indication that the pragmatic function of the nod+VSU composites is reducible to that of their component modalities.

We close this section with a note on “planning.” Above we have voiced certain reservations toward the notion of respiratory planning. However, a careful reader might point out that the view espoused in the present paper could also be subsumed under the very same rubric. Indeed, producing a short feedback expression on residual air or the choice of a nod in place of a verbal feedback expression could be considered a case of linguistic planning. It is, however, planning of a radically different kind. Consider for instance a classic model by Levelt (1993), in which speech planning is executed sequentially with no feedback loops between the components. Accordingly, in Levelt's model there is no way in which current respiratory state could influence the choice of non-verbal rather than verbal feedback at low lung volumes as this decision would need to be made at a very early planning step when no access to any kind of sensory feedback is available. By contrast, our results suggest a stronger role played by proprioception in speech planning. In fact, it seems that no context-free, symbolist model which does not put embodiment at its core can account for the data. In other words, we postulate a system in which “feedback would not necessarily be limited to the sequencing of movements but rather would be important in the shaping of movements” (Löfqvist, 2010, p. 407)

In addition, we suggest that ensuring sufficient amount of air in the lungs is only *one of several* of speaker's goals in a regular conversation. As suggested by Horii and Cooke (1978), speakers always have the option of speaking below REL, which although suboptimal from the point of view of the production system, is a perfectly viable production strategy and could be favored for pragmatic reasons, such as temporal organization of conversation, or to accommodate to a contemporaneous task, which might require reorganization of the speech-breathing coordination (cf. Fuchs et al., 2015b). From that perspective, also the economy principle is just one of several strategies available to the user and can be overridden by other externalities. Or to quote Lindblom (1990) again, “[i]f the speech system operates so as to minimize “articulatory effort” […], we should expect it to undershoot phonetic targets quite often, but not necessarily in every single instance. The key point is: *Speakers have a choice*. [emphasis in the original].”

Lastly, it should be borne in mind that the motoric program itself need not be fully specified. Indeed, respiratory control seems to obey coordinative and compensatory properties of dynamical systems (Newsom Davis and Sears, 1970; Hayashi et al., 2005) suggesting that details of speech-breathing coordination might be resolved automatically without resorting to high cognitive functions. From the point of view, the volume-dependent pre-speech respiratory adaptations reported by McFarland and Smith (1992) need not, as Winkworth et al. (1994) insist, suggest “the existence of a planning function” (p. 554) as the observation is fully consistent with an account based on low-level automatic compensatory activity brought about by emergent and task-dependent coordinative structures (Kelso et al., 1980), in which the details of motor control depend on the current state of the system (Löfqvist, 2010).

In conclusion, the present paper is but a small attempt at explaining the complex interactions between speech and breathing. Specifically, we demonstrated that by including phenomena specific to spontaneous conversation, such as verbal and non-verbal feedback expressions, we can gain deeper understanding of the underlying processes than relying on read and tightly controlled lab setups can provide. Indeed, the tight control in some of the earlier paper on respiratory planning in speech would make any of our observations impossible. For instance, in order to separate the effects of the previous utterance, Whalen and Kinsella-Shaw (1997) had their participants initiate an inhalation at the same constant lung volume for each utterance. While that is a valid methodological technique, its ecological validity is limited and it prevents observing any other complementary mechanisms which might be at play when speech breathing is studied in its natural context.

We hope to have demonstrated that much is to be gained by looking closely at spontaneous conversations. In this particular case, coordination of speech and nod onsets with respect to the respiratory cycle suggests existence of temporal patterns consistent with an economy principle. In short, within the limits of their communicative goals (e.g., producing feedback) speakers seem to adapt their behavior in such a way that respiratory effort (i.e., the need for a new respiratory cycle) is minimized. Consequently, communicative needs, respiratory constraints and momentary lung volume jointly shape the coordinative respiratory patterns observed in speech.

## Author contributions

MW, MH: The conception and design of the work; acquisition, analysis and interpretation of the data; drafting the work; final approval of the version to be published.

### Conflict of interest statement

The authors declare that the research was conducted in the absence of any commercial or financial relationships that could be construed as a potential conflict of interest. The reviewer EB and handling Editor declared their shared affiliation, and the handling Editor states that the process nevertheless met the standards of a fair and objective review.
